# CD19: a biomarker for B cell development, lymphoma diagnosis and therapy

**DOI:** 10.1186/2162-3619-1-36

**Published:** 2012-11-29

**Authors:** Kemeng Wang, Guoqing Wei, Delong Liu

**Affiliations:** 1 Division of Hematology and Oncology, Department of Medicine, New York Medical College and Westchester Medical Center, Valhalla, NY 10595, USA; 2 Bone Marrow Transplantation Center, the First Affiliated Hospital, Zhejiang University School of Medicine, Hangzhou, China

## Abstract

The human CD19 antigen is a 95 kd transmembrane glycoprotein belonging to the immunoglobulin superfamily. CD19 is classified as a type I transmembrane protein, with a single transmembrane domain, a cytoplasmic C-terminus, and extracellular N-terminus. CD19 is a biomarker for normal and neoplastic B cells, as well as follicular dendritic cells. CD19 is critically involved in establishing intrinsic B cell signaling thresholds through modulating both B cell receptor-dependent and independent signaling. CD19 functions as the dominant signaling component of a multimolecular complex on the surface of mature B cells, alongside complement receptor CD21, and the tetraspanin membrane protein CD81 (TAPA-1), as well as CD225. Through study of CD19 transgenic and knockout mouse models, it becomes clear that CD19 plays a critical role in maintaining the balance between humoral, antigen-induced response and tolerance induction. This review also summarized latest clinical development of CD19 antibodies, anti-B4-bR (an immunotoxin conjugate), blinatumomab (BiTE), and SAR3419 (huB4-DM4), a novel antibody-drug conjugate.

## 

Rituximab, the first monoclonal antibody against CD20 molecule, has revolutionized lymphoma therapy [1,2]. More monoclonal antibodies targeting different antigens are being developed for lymphoma therapy [3]. CD19 is specifically expressed in normal and neoplastic lymphoid cells. This review summarizes the molecular structure and functions of CD19 antigen as well as the clinical development of CD19 monoclonal antibodies for lymphoma therapy.

## CD19 gene and antigen

**Figure 1:**
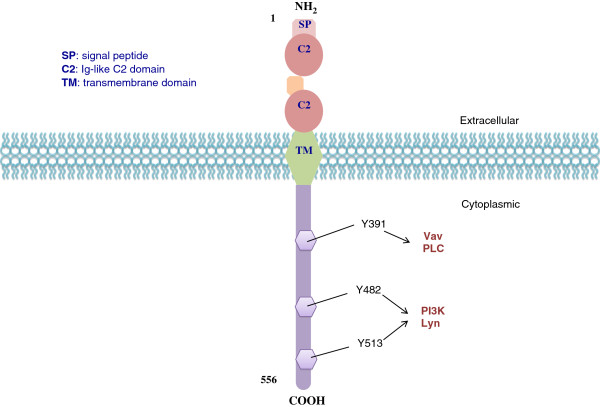
**CD19 molecular structure. **CD19 is a type I one-pass transmembrane protein. The two extracellular C2 Ig-like domains are separated by a small helical non-Ig domain with possible disulfide links. The highly conserved, 242 amino acid cytoplasmic domain includes multiple tyrosine residues. Three key tyrosine residues are shown with their associated signaling kinases and molecules.

The human CD19 antigen is a 95 kd transmembrane glycoprotein belonging to the immunoglobulin (Ig) superfamily [4,5]. It is encoded by the 7.41 kilobite *cd19* gene located on the short arm of chromosome 16, 16p11.2 [6]. The gene contains 15 exons and codes for the CD19 molecule with 556 amino acids (Figure 1). There are more than one mRNA transcripts, though only two transcript isoforms have been isolated *in vivo*[5-7]*.* Structurally, the gene contains an unusually short 5'-untranslated region. The proximal *cd19* promoter lacks a TATA box, and its major start sites are found within 50 bp of the initiation codon [8].

**Figure 2:**
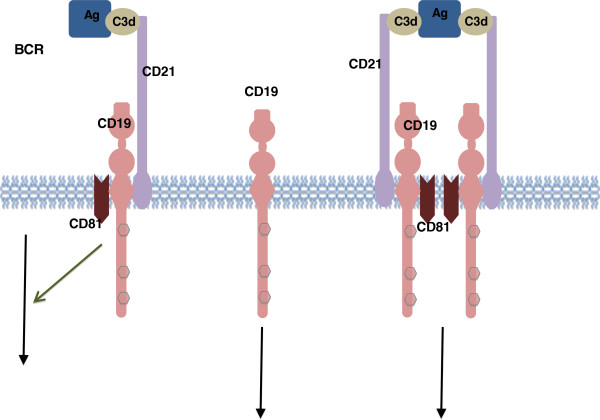
**CD19 associated signaling complex. **Antigen-C3d complexes can engage the CD19/21 complex in both a BCR-independent or BCR-dependent fashion. The CD19 complex includes complement receptor CD21, which binds C3d-modified antigen.

CD19 is classified as a type I transmembrane protein, with a single transmembrane domain, a cytoplasmic C-terminus, and extracellular N-terminus. No significant homology exists between CD19 and other known proteins [9]. The extracellular element contains two C2-type Ig-like domains divided by a smaller potential disulfide linked non-Ig-like domain, as well as N-linked carbohydrate addition sites (Figure 2). The highly conserved cytoplasmic domain consists of 242 amino acids with nine tyrosine residues near the C-terminus [9-11]. Multiple studies have come to suggest that the biologic functions of CD19 are dependent on three cytoplasmic tyrosine residues – Y391, Y482 and Y513. Experiments have shown that substitution of phenylalanine for tyrosine at two of the positions, Y482 and Y513, leads to inhibited phosphorylation of the other seven tyrosines [12-14].

## Kinetics of CD19 expression

CD19 was first identified as the B4 antigen of human B lymphocytes through the use of anti-B4 monoclonal antibody (mAb) against CD19. It is specifically expressed in normal and neoplastic B cells, as well as follicular dendritic cells [9,11,15]. During B cell lymphopoiesis, the surface expression of CD19 first takes place around the time of immunoglobulin gene rearrangement [9]. During this process, Pax5 is required for the normal expression of CD19. This was proven by the fact that lymphoid progenitors in Pax5 knockout mice arrest at the pro-B cell stage. The surface density of CD19 is highly regulated throughout B cell development and maturation, until the loss of expression during terminal plasma cell differentiation [9,11]. CD19 expression in mature B cells are 3-fold higher than that found in immature B cells, with slightly higher expression in B1 cells than in B2 (conventional B) cells [11,12]. CD19 is one of the most reliable surface biomarker for B cells. It is expressed from pre-B cells until the terminal differentiation to plasma cells.

## Physiological function

CD19 is critically involved in establishing intrinsic B cell signaling thresholds through modulating both B cell receptor (BCR)-dependent and independent signaling [16,17]. It plays roles in the antigen-independent development as well as the immunoglobulin-induced activation of B cells. CD19 is thus critical for the body to mount an optimal immune response*.* CD19 works in complex with the BCR and other surface molecules to allow both direct and indirect recruitment and binding of various down-stream protein kinases [6,18]. The protein kinases that interact with the CD19 complex include those belonging to the Src family (Lyn, Fyn), Ras family, Abl, Btk, adapter molecules (Vav, Grb2), and PI3K [9]. More recently, it has been recognized that CD19 is required for optimal MHC class II-mediated signaling, through its modulation of tyrosine phosphorylation and Akt kinase signaling [19].

CD19 functions as the dominant signaling component of a multimolecular complex on the surface of mature B cells, alongside complement receptor CD21 (CD2), and the tetraspanin membrane protein CD81 (TAPA-1), as well as CD225 [9,13,18,20] (Figure 2). The CD19 complex functions to decrease the threshold for receptor-dependent signaling through modulating both intrinsic and receptor-induced signals [11,12,18]. CD19 acts as a critical co-receptor for BCR signal transduction [13,21]. As BCR signaling requires protein tyrosine kinase (PTK) activation, CD19 recruits and amplifies the activation of Src-family protein tyrosine kinases such as Lyn and Fyn [9,13,22]. Upon BCR activation, CD19 also enhances BCR-induced signaling crucial for B cell expansion, through recruitment and activation of PI3K and downstream Akt kinases [23].

The CD19/CD21 complex is also capable of, independent of the BCR, reacting and binding to activated complement fragment C3d. This complex subsequently translocates into membrane “lipid rafts” domains, where tyrosine phosphorylated CD19 can then interact with co-localized kinases in the membrane to modulate BCR signaling [24]. Normally, more CD19 is found associated with CD21 than in complex with BCR, and a dramatic increase in B-cell activation results from the simultaneous binding of surface antigen by B-cell Ig and of C3d by CD21. Studies also showed that CD19 does not require CD21 for signal transduction, and CD19/21 complex signaling is induced by the binding of C3d to CD21 [13,14].

CD81 functions as a part of the tetraspanin web as well as a chaperone protein. It provides docking sites for molecules involved in various signal transduction pathways, and is important for the expression of CD19. CD19 expression in B cells of CD81−/− mice were found to be ~30–50% of that in wild type (WT) mice, while expression of CD21 and the BCR were at their respective WT levels [25,26]. CD19/CD38 have been shown to co-localize in lipid rafts and physically interact with another surface antigen receptor, CD38, in mice B cell. The two receptors have been shown to be a part of the B cell signaling complex [24,25]. *In vivo*, CD81-deficient mice show reduced CD19 expression and overall B cell signaling. In the absence of CD81 expression, CD19 expression is halved [11].

CD19 is thought to play duel roles in B cell activation. First, it functions as an adaptor protein to recruit cytoplasmic signaling proteins to the membrane. Experiments have shown that CD19 tyrosine residues are phosphorylated. The tyrosine phosphorylated CD19 can recruit SH2 domain–containing cytosolic proteins after ligation with the BCR. The second role of CD19 is as a signal subunit for the CD19/CD21 complex when colligated with the BCR, where Ag bearing complement enhances B-cell activation via BCR-CD19/CD21 coligation [4,12,21,27].

In addition, CD19 is involved with, though not essential, in the regulation of bone marrow development through its actions on bone marrow cells via altering BCR signals [12]. Others have proposed a critical role for CD19 in the process of B cell development from their early differentiation events in the bone marrow to late maturation steps in the spleen [13]. In developing B cells, CD19 is capable of modulating signal thresholds independent of the BCR. In fact, CD19 signaling may play a role in controlling the progression of early pre-B to small, resting pre-B cells in the bone marrow by associating with components of the pre-BCR.

## CD19 transgenic mice (hCD19TG)

CD19 overexpression has been studied through use of hCD19TG mice which express a human CD19 transgene. Phenotypic analysis of hCD19TG mice show that CD19 overexpression is associated with a dramatic reduction (>80%) in the number of peripheral B cells, primarily due to impaired generation and early development of immature precursors in the bone marrow.

B cells of hCD19TG show a gene dosage dependent increase in proliferation in response to mitogens such as lipopolysaccharide (LPS), with homozygotes showing a much more dramatic increases in proliferation than that seen in CD19TG+/− heterozygous mice. B cells from hCD19TG +/+ mice also showed higher background proliferation, and survived longer than control cells in culture without any mitogen stimulation.

Although hCD19TG mice have significantly reduced numbers of peripheral B cells, they show an overall increase in serum immunoglobulin levels (~40% increase), but skewed responses of different immunoglobulin isotypes: IgG2b levels increased by 168%, IgG3 levels decreased by 77%, and IgA levels remaining relatively unchanged. CD19 overexpression therefore increases B cell sensitivity to transmembrane signaling and augments the overall susceptibility of B cells to induced differentiation. The increase in B cell surface receptor expression may feedback to inhibit the development of bone marrow B precursors, linking overexpression to the observed decrease in peripheral B cells.

## CD19 −/− mice

In contrast to hCD19TG mice, CD19 deficient mice is associated with defects in later stages of B cell growth and maturation that take place in the spleen and peripheral lymph tissues. CD19 deficiency had no significant effect on the number of B cell precursor in the bone marrow, nor on the size and morphology of B cells. CD19−/− mice instead show dramatic reductions in the total number and frequency of peripheral and splenic B cells, especially of B1 cells, a B cell subtype found in the peritoneal cavity that normally express higher IgM levels than conventional B ( B2) cells, and low levels of IgD. The B cells obtained from the peritoneum of CD19 −/− mice predominantly expressed high levels of both IgM and IgD.

The proliferative capacity of B cells in CD19 −/− mice is consistently reduced in response to stimulation by a range of LPS (mitogen) concentration, as well as in response to antibody cross-linking of surface IgM. CD19 is not required for clonal expansion, and B cells in CD19 deficient mice are able to proliferate and differentiate into plasma cells, albeit at reduced rates, consistent across all mitogen concentrations tested.

Unlike the skewed response of Ig isotypes in hCD19TG mice, levels of all immunoglobulin isotypes were significantly reduced in the CD19 −/− mice. The overall 77% reduction in Ig levels is primarily contributed to changes in the frequency of B cell differentiation, not the decrease of peripheral B cell numbers. In particular, the dramatic reduction of IgG1 (86% decrease) and IgG2a (84% decrease) hints that interactions between B cell and T cell, which facilitate these Ig isotypes, are particularly affected in CD19 deficiency. The reduced susceptibility to transmembrane signaling and lowered rates of proliferation in CD19 −/− mice leads to decreased formation of second follicles, germinal centers, and overall reduced ability of effective humoral immunity in response to T cell dependent antigen [28]. In CD19-deficient mice, loss of splenic marginal zone B cells has been observed, as well as significant deficiencies in specific peripheral B-cell subsets [12,13]. Furthermore, since CD19 is important both for B cell activation of T cell dependent antigen, as well as for B cell maturation and memory cell selection, mature CD19−/− B cells are profoundly deficient in responding to antigens that require T-cell help [13,27].

Selectively silencing CD19 expression by siRNA knockdown experiments leads not only to complete inhibition of CD19-mediated calcium fluxes, but also a total halt in CD38 signaling, without affecting the surface expression of CD38 [24,25]. These data provide additional support that CD19 is the main co-receptor in human B cells.

The critical importance has garnered CD19 the title of being a *rheostat* – for its crucial roles in the normal expansion and function of the peripheral B-cell pool [9]. It contributes to maintaining the balance between humoral, antigen-induced response and tolerance induction, as even small modulations in CD19 expression can impact B cell signaling thresholds and dramatically affect the sensitivity and specificity of B cell mediated immunity [14,28,29].

## Disease association

The importance of CD19 can be seen through case studies as well as various studies of CD19-deficient mice. CD19-deficient humans and mice exhibit hyporesponsiveness to transmembrane signals, and weak T cell-dependent humoral responses, leading to an overall impaired humoral immune responses [9,17]. Homozygous frame shift mutations of the *cd19* gene have been documented to result in truncation of the three key cytoplasmic tyrosine residues. Patients with this type of CD19 gene mutations showed normal numbers of precursor and total B cells, but decreased numbers of CD27+ memory B cells and CD5+ B cells. Their B cells exhibit normal levels of CD81 and CD225, but decreased CD21 and very low to undetectable CD19. The patients develop hypogammaglobulinemia, showing impaired antigen-induced BCR response and poor antibody response to rabies vaccination, as well as increased susceptibility to infection [18].

Studies showed that autoimmunity in CD19 transgenic mice depends on MHC class II expression [19]. This led to hypothesis that CD19 may play an important role in *in vivo* modulation of MHC class II expression and signaling. Such discoveries have led to mounting interest in CD19 as a potential immunotherapy target for various autoimmune disorders, including rheumatoid arthritis and multiple sclerosis.

Though it is not known if CD19 contributes directly to B cell carcinogenesis, its expression is highly conserved on most B cell tumors [17]. It is expressed in most acute lymphoblastic leukemias (ALL), chronic lymphocytic leukemias (CLL) and B cell lymphomas [30]. In fact, the majority of B cell malignancies express CD19 at normal to high levels (80% of ALL, 88% of B cell lymphomas and 100% of B cell leukemias) [9]. Other B cell malignancies, in contrast, show diminished CD19 levels [31,32]. Although CD19 expression is observed in normal plasma cells, malignant plasma (myeloma) cells isolated from multiple myeloma patients have been shown to lack CD19 expression, while isolates from pre-myeloma patients show a mix of both CD19- and CD19+ plasma cells [33]. CD19 levels can potentially be useful as a diagnostic tool in distinguishing certain lymphoma subtypes. Follicular lymphoma, for example, has lower CD19 level more frequently than any other lymphoma subtypes. Low CD19 is also more common in CD10-positive than in CD10-negative diffuse large B cell lymphoma (DLBCL) [31].

Interestingly, CD19, though a B cell hallmark, has also been observed in cases of myeloid malignancies, including in 2% of AML cases. Rare cases of CD 19-expressing myeloblastic leukemia (AML-M2) in fact lack any myeloid surface antigens [34,35].

Recent studies have constructed one model of lymphomagenesis involving CD19 and the proto-oncogene *c-Myc*. A positive feedback pathway in which upregulated CD19 expression and phosphorylation, induced by constitutive c-Myc overexpression, serve to further promote and stabilize c-Myc signaling, whose downstream effectors include important cell cycle regulators like *cyclin D2*. Dysregulation in these regulators subsequently enhance lymphomagenesis. Using transgenic c-Myc mice, these studies have shown that CD19 expression, although not required for the malignant transformation in c-Myc–derived lymphomas, accelerates lymphomagenesis and is associated with increased disease severity. On the other hand, c-myc transgenic mice with CD19 deficiency, as compared to those expressing CD19, exhibited reduced malignancies and significantly higher (>80%) increase in survival and life spans [17].

## CD19 monoclonal antibodies for lymphoma and leukemia therapy

CD19 monoclonal antibodies have been explored for lymphoma therapy. Unconjugated mouse IgG2a anti-CD19 monoclonal antibody (mAb) was studied in six patients with progressive B cell lymphoma. The dosage ranged from 225 mg to 1000 mg. Transient reduction of tumor cells were seen. One patient achieved partial remission twice [36]. Following the study, the mAb was combined with interleukin-2 to treat 7 patients with low grade lymphoma. More tumor cell reduction was achieved in this study [37].

An immunotoxin conjugate of anti-B4-blocked ricin (Anti-B4-bR) was studied as adjuvant therapy for patients with B-cell non-Hodgkin's lymphoma (NHL) in complete remission (CR) after autologous bone marrow transplantation [38]. Forty-nine patients post-autologous stem cell transplantation received the immunotoxin at a dose of 30 microg/kg daily for 7 days by continuous i.v. infusion.Thirty one patients received two or more courses at 14-day intervals. The mean serum level on day 7 with the first course was found to be 0.77+/−0.41 nM. Twenty three patients developed human antimouse antibody and/or human anti-ricin antibody at a median of 22 days from the initiation of Anti-B4-bR therapy (range, 11–100 days).

A bi-specific T-cell engaging (BiTE®) antibody, blinatumomab, was engineered to direct cytotoxic T-cells to B-cells expressing CD19. An open-label, multicenter, single-arm, exploratory phase II trial was conducted to evaluate efficacy and safety of BiTE in adult patients with relapsed /refractory B-precursor ALL. Blinatumomab was given at 15 μg/m^2^/day as continuous intravenous infusion for 28-days followed by a 14-day rest. A total of up to 5 cycles of blinatumomab treatment was given to responding patients at one of three dose levels. A total of 18 patients were enrolled at the last update in 2011. Twelve out of 18 patients (RR 67%) have achieved a complete remission within the first 2 cycles. These responders included 3 patients with t(4;11) and 1 patient with Ph-positive B-precursor ALL. Four responders proceeded to allogeneic HSCT. Major adverse events included pyrexia, chills, disseminated intravascular coagulation /cytokine release syndrome leading to treatment discontinuation, as well as fully reversible CNS serious adverse events. Adding a cytoreductive pre-phase and implementing a lower initial dosing at 5 μg/m^2^/day in cohort 2a during the first week eliminated further treatment discontinuations. There were no treatment related deaths. A maintenance dose of 30μg/m^2^/day in cohort 2b increased the number of adverse events. Therefore, cohort 2a was selected for further clinical development in this patient population [39].

SAR3419 (huB4-DM4) is a novel antibody-drug conjugate [40]. A humanized IgG1 anti-CD19 monoclonal antibody was conjugated to a maytansine derivative DM4 through a cleavable disulfide-bond linker [41]. DM4, the maytansine derivative, is a potent tubulin inhibitor, which is specifically directed to CD19-positive B cells by the anti-CD19 mAb. A phase I, open-label, dose-escalation study of SAR3419 as a single agent was conducted as a first-in-human trial [42]. SAR3419 was administered intravenously every three weeks for up to six cycles to 39 patients with relapsed CD19+ B-cell lymphoma. Dose levels ranged from 10 to 270 mg/m^2^. The dose-limiting toxicities occurred in seven patients. These included severe blurred vision associated with microcystic epithelial corneal changes. The maximal tolerated dose (MTD) was 160 mg/ m^2^. Twenty-six patients (74%) showed tumor response; six of those patients achieved PR or CR. In particular, seven (47%) of 15 patients who had rituximab-refractory disease had tumor shrinkage. SAR3419 had an elimination half-life in the range of 3 to 7 days. Therefore, SAR3419 was shown to have a safe profile when administered to patients with relapsed B-cell lymphoma with the MTD at 160 mg/m^2^.

## Conclusion and future directions

CD19 is a biomarker for B cells. CD19 functions as the dominant signaling component of a multimolecular complex on the surface of mature B cells, alongside complement receptor CD21, and the tetraspanin membrane protein CD81 (TAPA-1), as well as CD225. CD19 plays a critical role in maintaining the balance between humoral, antigen-induced response and tolerance induction. Clinical development of CD19 monoclonal antibodies, anti-B4-bR, BiTE, and SAR3419 (huB4-DM4) are still in the early phase. It is forseeable that CD19 mAb will be widely studied for therapies of lymphoma, leukemia and autoimmune disorders. Through molecularly engineered chimeric antigen receptor, CD19 directed immunotherapy for refractory CLL is promising [43,44]. Further study in controlled trial with more patients will be critical to validate this approach.

## Competing interests

The authors have no relevant conflicts of interest.

## Authors’ contributions

All authors have contributed to data preparation, drafting and revising the manuscripts. All authors have read and approved the final manuscript.
